# Depression, sexual dysfunction and sexual quality of life in women with infertility

**DOI:** 10.1186/s12905-018-0584-2

**Published:** 2018-06-14

**Authors:** Zahra Shahraki, Fatemeh Davari Tanha, Mahsa Ghajarzadeh

**Affiliations:** 1 Zabol University of Medial Sciences, Zabol, Iran; 2 Department of Obstetrics and Gynecology, Yas Hospital, Tehran University of Medical Sciences, Nejatollahi street, Karimkhan, Tehran, Iran; 3 Universal Council of Epidemiology (UCE), Universal Scientific Education and Research Network (USERN), Imam Hospital, Tehran University of Medical Sciences, Tehran, Iran

**Keywords:** Sexual function, Quality of life, Depression, Iran, Infertility

## Abstract

**Background:**

Women suffering from infertility are at higher risk of experiencing psychological problems. Sexual function and sexual related quality of life is not considered as it should be. We designed this study to assess depression, sexual function and sexual quality of life in Iranian women with infertility.

**Methods:**

Two hundred and sixty four individuals participated in the study (115 healthy controls, 78 with primary and 71 with secondary infertility). All participants were asked to fill a valid and reliable Persian versions of BDI (Beck depression inventory), FSFIS (Female Sexual Function Index) and sexual quality of life-Female (SQOL-F) questionnaires.

**Results:**

Mean BDI score was significantly lower in healthy individuals.Individuals with primary infertility suffered more from sexual dysfunction, while BDI score was significantly higher and SQOL-F was significantly lower in cases with sexual dysfunction. There was significant positive correlation between SQOL-F and total FSFI score (*r* = 0.59, *p* < 0.001). Linear regression analysis by considering SQOL-F as dependent and age, BDI, duration of marriage, sexual dysfunction (FSFI ≤26.55 or > 26.55) showed that BDI and sexual dysfunction were independent predictors of SQOL-F.

**Conclusion:**

Sexual function and quality of life related to sexual life should be considered in Iranian infertile ones. Depression as a crucial factor should be focused more in infertile women.

**Electronic supplementary material:**

The online version of this article (10.1186/s12905-018-0584-2) contains supplementary material, which is available to authorized users.

## Background

Inability to conceive after one year of sex without protection is considered as infertility that involves one out of five couples [1]. Infertility is a stressful condition that affects all aspects of affected couple’s life [2, 3]. Impaired mental, physical, social and personal aspects of life along with waste of time and money, marital disruption and divorce [1]. Almost 40% of couples who seek infertility treatment still cannot conceive [4]. Psychological problems such as depression, anxiety, stress, suicidal attempt are more common in infertile women [2, 3]. Literature show that sexual dysfunction is higher in infertile couples in comparison with healthy controls [5, 6]. Although sexual function is one of the key factors in physical and marital health, it is not considered as it should be. Different factors have been deliberated to influence sexual function in women with infertility such as age, psychological status, adverse effects of medications, and sex hormone status [1].

Recently some studies conducted to evaluate sexual function in these women and differentiate between sexual statuses of primary or secondary infertile cases but there is no study evaluating quality of sex in this group.

We designed this study to assess depression, sexual function and sexual quality of life in Iranian women with infertility.

## Methods

This cross sectional study conducted in Women’s hospital (affiliated hospital of Tehran university of medical sciences) between March 2015 and March 2017.

Inclusion criteria were: Inclusion criteria were: age more than 18 and less than 40, candidates for IVF and no underlying diseases such as kidney disease, coronary disease, PCO (poly cystic ovarian syndrome). All cases were asked to fill informed consent forms before study.

Healthy subjects (from hospital workers) with above inclusion criteria and no history of infertility enrolled in the study.

They also were asked to fill valid and reliable Persian versions of BDI (Beck depression inventory), FSFIS (Female Sexual Function Index) and sexual quality of life-Female (SQOL-F) questionnaires.

BDI consists of 21 questions; each has scores between 0 and 3. Scores between 0 and 9 is indicative of not-depressed individuals, scores between 10 and 18 is indicative of mild, 19–29 moderate and > 29 severe depression [7].

FSFI includes 19 questions, evaluating sexual function in six domains (desire, arousal, lubrication, orgasm, satisfaction and pain. Total score shows sexual function [8]. Lower score is indicative of more sexual dysfunction. The cutoff point of 26.55 is considered to determine sexual dysfunction.

SQOL-F is a self-report questionnaire assessing the impact of sexual dysfunction. It measures sexual confidence, emotional well-being, and relationship issues. It includes 18 questions, each containing a six-point likert scale answers (completely agree to completely disagree). The total score is sum of total scores of questions. Higher the score, higher the quality of life [9].

SPSS version 24 (SPSS Inc., Chicago, IL, USA) was used for data analysis. Data was shown in mean ± SD. Independent sample t test and paired sample t test applied for continuous, as well as the Pearson X^2^ test with Fisher’s exact test used for assessment of categorical variables. Correlation coefficient was applied. Linera regression analysis conducted.

*P-*value < 0.05 was considered statistically significant.

## Results

Two hundred and sixty four individuals participated in the study (115 healthy controls, 78 with primary and 71 with secondary infertility). Partner age, duration of marriage and history of abortion were significantly different between groups (Table 1).

**Table 1:** Basic characteristics of groups

	Healthy subjects	Primary infertility	Secondary infertility	*P* value
Age (year)	32.9 ± 7.2	31.3 ± 6.2	32.4 ± 5.4	0.2
Partner age (year)	37.7 ± 8.3	34.2 ± 6	37.2 ± 5.8	0.003
Duration of marriage (year)	11.1 ± 7.9	6.2 ± 4	10.1 ± 5.2	<0.001
Previous abortion	33(29.7%)	1(1.3%)	46(65.7%)	<0.001

BDI score was significantly lower in healthy subjects (Table 2).

**Table 2:** Comparison of BDI, FSFI domains and SQOL-F in three groups

	Healthy subjects	Primary infertility	Secondary infertility	*P* value
BDI	13 ± 9.1	16.3 ± 8.7	16 ± 10.4	0.02
Desire	3.8 ± 1.1	3.9 ± 1	3.7 ± 0.9	0.6
Arousal	3.9 ± 1.4	3.8 ± 1	3.8 ± 1.1	0.6
Lubricant	4.4 ± 1.5	4.3 ± 1.2	4.5 ± 1.2	0.6
Orgasm	4.5 ± 1.5	4.1 ± 1.3	4.4 ± 1.1	0.09
Satisfaction	4.7 ± 1.2	4.4 ± 1.3	4.6 ± 1.1	0.3
Pain	4.2 ± 1.4	4.1 ± 1.2	4.3 ± 1.3	0.6
Total FSFI	25.7 ± 7.2	24.7 ± 5.1	25.2 ± 5.3	0.5
SQOL-F	84.8 ± 18.9	83.4 ± 17	80.1 ± 17.6	0.2

Individuals with primary infertility suffered more from sexual dysfunction, while BDI score was significantly higher and SQOL-F was significantly lower in cases with sexual dysfunction (Table 3).

**Table 3:** Comparison of number of cases, BDI and SQOL-F in two groups: with and without sexual dysfunction

	FSFI> 26.55	FSFI≤26.55	*P* value
Group			
Healthy subjects	51(44.3%)	64(55.7%)	0.01
Primary infertility	51(65.4%)	27(34.6%)	
Secondary infertility	37(52.1%)	34(37.9%)	
BDI	18 ± 9.5	11.3 ± 8.1	< 0.001
SQOL-F	73.4 ± 16.5	94.1 ± 12.8	< 0.001

There was significant positive correlation between SQOL-F and total FSFI score (*r* = 0.59, *p* < 0.001) and also significant negative correlation between SQOL-F and BDI (*r* = − 0.49, *p* < 0.001).

Linear regression analysis by considering SQOL-F as dependent and age, BDI, duration of marriage, sexual dysfunction (FSFI ≤26.55 or > 26.55) showed that BDI and sexual dysfunction were independent predictors of SQOL-F (Table 4).

**Table 4:** Linear regression analysis by considering SQOL-F as dependent variable

	β	*P* value
Age	−0.08	0.6
Duration of marriage	−0.03	0.8
BDI	−0.6	< 0.001
Sexual dysfunction	16.3	< 0.001

## Discussion

To our knowledge this is the first study evaluating sexual quality of life in infertile women in Iran.

The results showed that sexual dysfunction is more common in women with primary infertility and BDI score was significantly higher in individuals with sexual dysfunction. The results also showed that total FSFI score and its domains are not significantly different between infertile subjects and healthy ones. These findings are compatible with Monga et al. findings. They compared 18 infertile couples and 12 healthy ones and reported no significant difference between sexual function of fertile vs infertile groups [4]. In our previous study, we only assessed sexual function in infertile women with primary or secondary infertility in comparison with controls. Our results showed that total FSFI score and all of its domains were significantly higher in controls vs. infertile ones while only desire subscale was significantly higher in primary group [1].

By comparing secondary and primary infertility groups of women, Keshin et al. stated that orgasm, satisfaction domain of FSFI and total FSFI scores were significantly lower in secondary groups and sexual dysfunction in secondary group after adjustment for confounders was 9.5 fold of primary group [10].

In current study, sexual dysfunction was significantly higher among women with primary infertility in comparison with secondary group and healthy ones.

In a previous study, Millheiser et al. found sexual dysfunction in 25% of fertile group and 40% of infertile women and against our findings, desire, arousal and satisfaction domains were significantly higher in fertile group [5].

Studies conducted on infertile women, mostly focused on general quality of life and did not considered sexual quality of life. In a recent study by Aduloju et al.,

Quality of life score of fertile women was significantly higher than infertile individuals and women with secondary infertility had overall higher scores [11].

In another study in Turkey, SEZGIN et al. reported worse quality of life in infertile women than fertile ones [12].

By means of FSFI and core Fertility Quality of Life questionnaires, Lo and Kok found that infertile women with sexual dysfunction had lower quality of life score [13].

Our results show that SQOL-F score was significantly lower in cases with sexual dysfunction and there was significant positive correlation between FSFI and SQOL-F scores.

Linear regression analysis also showed that BDI was a negative independent of SQOL-F while was a positive independent predictor.

Although most of the time it is neglected, sexual health affects general health and sexual problems negatively influence quality of life [14].

Literature shows that sexual dysfunction in patients with various diseases will result in impaired quality of life [15–17].

SQOL includes questions assessing thoughts and feelings about sexual life containing sexual participation concern and sexual function perception [9].

Sexual confidence, emotional well-being, and relationship issues are evaluated by means of SQOL [18]. As it is presented, total FSFI and SQOL scores are moderately correlated which could be indicative of assessing related concepts which is compatible with Pakpour et al. findings [9].

It should be considered that infertility affects different aspects of couple’s lives including psychological well-being. Between 40 and 50% of infertile women experience emotional problems [19]. Depression, anxiety, marital dis-adjustment, sexual dysfunction and impaired quality of life reported in more women with infertility [1, 4]. The most prevalent psychological problem in infertile women is depression which is more common in cases with primary infertility [10]. Our results show that mean BDI score was significantly lower in healthy subjects than infertile ones.

This study has some advantages. First, it is the first study evaluating sexual quality of life in Iranian women with infertility. Second, we included either individuals with primary or secondary infertility as well as healthy controls.

This study had also limitations. First, it conducted in a tertiary center. Second, no comprehensive information of partners were available. Multi centric studies with large sample sizes are recommended.

## Conclusion

Sexual function and quality of life related to sexual life should be considered in Iranian infertile ones. Depression as a crucial factor should be focused more in infertile women.

## Additional file

Additional file 1: SPSS. Data used for analysis in this study. (SAV 28 kb)

## Abbreviations

BDI: Beck depression inventory
FSFIS: Female Sexual Function Index
PCO: poly cystic ovarian syndrome
SQOL-F: Sexual quality of life-Female

## References

[1] Tanha FD, Mohseni M, Ghajarzadeh M (2014). Sexual function in women with primary and secondary infertility in comparison with controls. Int J Impot Res.

[2] Baldur-Felskov B, Kjaer S, Albieri V, Steding-Jessen M, Kjaer T, Johansen C (2012). Psychiatric disorders in women with fertility problems: results from a large Danish register-based cohort study. Hum Reprod.

[3] Kjaer TK, Jensen A, Dalton SO, Johansen C, Schmiedel S, Kjaer SK (2011). Suicide in Danish women evaluated for fertility problems. Hum Reprod.

[4] Monga M, Alexandrescu B, Katz SE, Stein M, Ganiats T (2004). Impact of infertility on quality of life, marital adjustment, and sexual function. Urology.

[5] Millheiser LS, Helmer AE, Quintero RB, Westphal LM, Milki AA, Lathi RB (2010). Is infertility a risk factor for female sexual dysfunction? A case-control study. Fertil Steril.

[6] Ercan C, Coksuer H, Aydogan U, Alanbay I, Keskin U, Karasahin K (2013). Sexual dysfunction assessment and hormonal correlations in patients with polycystic ovary syndrome. Int J Impot Res.

[7] Ghassemzadeh H, Mojtabai R, Karamghadiri N, Ebrahimkhani N (2005). Psychometric properties of a Persian-language version of the Beck depression inventory-second edition: BDI-II-PERSIAN. Depress Anxiety.

[8] Fakhri A, Pakpour AH, Burri A, Morshedi H, Zeidi IM (2012). The female sexual function index: translation and validation of an Iranian version. J Sex Med.

[9] Pakpour AH, Zeidi IM, Saffari M, Burri A (2013). Psychometric properties of the Iranian version of the sexual quality of life scale among women. J Sex Med.

[10] Keskin U, Coksuer H, Gungor S, Ercan CM, Karasahin KE, Baser I (2011). Differences in prevalence of sexual dysfunction between primary and secondary infertile women. Fertil Steril.

[11] Aduloju OP, Olaogun OD, Aduloju T (2017). Quality of life in women of reproductive age: a comparative study of infertile and fertile women in a Nigerian tertiary Centre. J Obstet Gynaecol.

[12] Sezgin H, Hocaoglu C, Guvendag-Guven ES (2016). Disability, psychiatric symptoms, and quality of life in infertile women: a cross-sectional study in Turkey. Shanghai Arch Psychiatry.

[13] Lo SS, Kok WM (2016). Sexual functioning and quality of life of Hong Kong Chinese women with infertility problem. Hum Fertil.

[14] Malouf MA, Inman AG, Carr AG, Franco J, Brooks LM (2010). Health-related quality of life, mental health and psychotherapeutic considerations for women diagnosed with a disorder of sexual development: congenital adrenal hyperplasia. Int J Pediatr Endocrinol.

[15] Naeinian MR, Shaeiri MR, Hosseini FS (2011). General health and quality of life in patients with sexual dysfunctions. Urol J.

[16] Tepavcevic D, Kostic J, Basuroski I, Stojsavljevic N, Pekmezovic T, Drulovic J (2008). The impact of sexual dysfunction on the quality of life measured by MSQoL-54 in patients with multiple sclerosis. Mult Scler J.

[17] Di Fabio F, Koller M, Nascimbeni R, Talarico C, Salerni B (2008). Long-term outcome after colorectal cancer resection. Patients' self-reported quality of life, sexual dysfunction and surgeons' awareness of patients' needs. Tumori.

[18] Symonds T, Boolell M, Quirk F (2005). Development of a questionnaire on sexual quality of life in women. J Sex Marital Ther.

[19] Wallach EE, Seibel MM, Taymor ML (1982). Emotional aspects of infertility. Fertil Steril.

